# The effect of shared leadership on employee resilience: wielding the double-edged sword

**DOI:** 10.3389/fpsyg.2025.1441660

**Published:** 2025-02-20

**Authors:** Ma Hao, Yan Ai Min, Liu Jie, Deng Yuling

**Affiliations:** ^1^School of Business, Central South University, Changsha, Hunan Province, China; ^2^Department of Health Management Center, Third Xiangya Hospital, Central South University, Changsha, Hunan Province, China

**Keywords:** shared leadership, employee resilience, flexible work arrangements, role overload, goal clarity, job demands-resources model

## Abstract

**Introduction:**

This investigation delves into the ambidextrous impacts that shared leadership imparts on the resilience of employees through the incorporation of the Job Demands-Resources framework to delineate the underlying mechanisms. Furthermore, it explores the organizational boundary factors that enhance employees' resilience, so as to explore the ways to improve employees resilience.

**Methods:**

This research chose to use an online questionnaire at two time points with a analysis of 246 valid questionnaires. Surveys were disseminated electronically through esteemed third-party survey platforms so jump.

**Results:**

Empirical findings illustrate that SLP fosters resilience by bolstering FWA while concurrently heightening role overload and hampering resilience. Furthermore, the study examines how goal clarity modifies these conflicting outcomes. Empirical evidence corroborates the positive moderating role of goal clarity in enhancing the influence of SLP on FWA, and the hypothesized moderating impact of goal clarity on the association between SLP and employees' perception of role overload is also affirmed.

**Discussion:**

This research contributes to the scholarly discourse by illuminating a more comprehensive understanding of the relationship between shared leadership and employee resilience, and further deepens insights into the moderating factors influencing employees resilience by assessing the moderating effect of goal clarity. It also offers practical insights into implementing shared leadership in a manner that harnesses its potential while mitigating its downsides on employee resilience.

## 1 Introduction

Companies now are facing a more volatile, uncertain, complex, and ambiguous competitive environments (Worley and Jules, 2020). Increasing numbers of practices and research cases show that the traditional centralized leadership style, with a single individual as the leader, can no longer adapt to the existing competitive environment (Ali et al., 2020), and the leadership style in team management is in urgent need of “transformation and upgrading,” notably during the unprecedented challenges (Fernandez and Shaw, 2020). In response, scholars have increasingly advocated for the reorientation of the conventional leadership model, positing collaborative leadership as a mechanism to amplify team effectiveness (Bövers and Hoon, 2020). Furthermore, there is an inclination toward decentralizing traditional leadership roles and distributing leadership responsibilities across the entire team (Hiller et al., 2006). These views coincide with the essence of shared leadership (SLP; Edelmann et al., 2020), which means a dynamic property within a team and characterized by the collective exercise of leader roles in collaborative manner (Carson et al., 2007). This leadership style no longer focuses on the characteristics of a certain individual in a team, but involves a dynamic and interactive relationship (Sinha et al., 2021).

It is revealed SLP could promote organizational performance (Cooper et al., 2014) and strategic performance at the organizational level (Yu et al., 2022), positively predicted team creativity (Ali et al., 2023), team proactivity (Erkutlu, 2012), team innovation (Hoch, 2013; Liang et al., 2021), team effectiveness (Choi et al., 2017), team adaptability (Burke et al., 2003), and could actively stimulate innovative behavior (Hoch, 2013), proactive behaviors (Fu et al., 2020) and so on at the employee level. However, there is a lack of sufficient attention to the individual capability (Liang et al., 2021), especially ignoring the capability of productively responding to changes and setbacks in their work under this VUCA context. What' more, even the inspiration for the effectiveness of SLP once made many researchers and management practitioners sound the “trumpet of cheer,” the negative effects of this kind of leadership are gradually “coming to the surface.” Previous research indicated that SLP can reduce individual creativity (Wang and Peng, 2022) and team performance (Han et al., 2021), while comparing to favorable effects attributed to SLP on organizations and individuals, research on its negative impact is still relatively insufficient. A lack of awareness regarding the potential drawbacks associated with SLP impedes a thorough appreciation of its complex effects on the workforce (Wang and Peng, 2022), but may also pose potential risks for the development of enterprises in the large-scale implementation of SLP. Academia has advocated for an expanded exploration of the adverse aspects of SLP (Zhu et al., 2018). Therefore, this study aimed to delve into the nuanced influences—both advantageous and adverse—of SLP on employee resilience.

The concept of employee resilience refers to the capacity of employees to effectively manage work-related changes and adversities, and subsequently adjust and prosper in new conditions (Caniëls and Hatak, 2022). The contemporary connotation of employee resilience focuses on engaged employee behaviors when facing large-scale crises, as well as changeable and uncertain challenges (Caniëls and Baaten, 2019). Individual capability is essential for the sustainable development of organizations (Mui Hung Kee and Chung, 2021). Considering this significant employee resilience's impact on individuals as well as organizations (Santoro et al., 2021), and the limited exploration of negative leadership factors related to employee resilience, our analysis centers on the paradoxical effects of SLP concerning the resilience of employees. Employee resilience could help individuals effectively cope with changing environments and adversity (Rossi et al., 2013), reduce psychological stress (Chen et al., 2017) and workplace stress (Badu et al., 2020), and buffer against burnout caused by stress (Dunn et al., 2008; Malik and Garg, 2020). Employee resilience can also help employees engage more with work (Cooke et al., 2019) and lead to more organizational resilience (Santoro et al., 2021), all of which have caused employee resilience to receive extensive attention from organizations (Hartmann et al., 2020).

The modern view acknowledges that employee resilience is product of person-environment interaction (Kuntz et al., 2016) and is shaped by the environment (Stokes et al., 2019). Thus, the workplace context is an antecedent factor that enhances or weakens employee resilience (Vera et al., 2017). Among these antecedent variables, leadership style is a key workplace context that affects employee resilience (Franken et al., 2020). Considering SLP can reduce employee creativity (Wang and Peng, 2022), and its active promotion effect on proactive employee behaviors (Fu et al., 2020), voice behavior (Ning and Hui, 2020), it is conceivable SLP could have dual impacts on employee resilience.

The Job Demands-Resources (JD-R) model is bifurcated into the components of job resources and job demands (Bakker and Demerouti, 2007; Bakker et al., 2003; Demerouti et al., 2001), and the emergence of motivational developments alongside job-related strain is steered by two psychological pathways: The motivational process and health impairment process (Schaufeli et al., 2009). To be more specifically, SLP can empower team members with more of a leader role (Shane Wood and Fields, 2007), emphasize overall coordination and the exchange of information in a team (Nordbäck and Espinosa, 2019), and encourage mutual support and care among members (Nazarpoori, 2017), all of which allow team members to participate flexibly and make autonomous decisions on their work arrangements, leading to a higher level of flexible work arrangements (FWA). FWA is a typical job resource (Kelly et al., 2020; Yeves et al., 2022) that not only promotes the improvement of individual resources (Duncan and Pettigrew, 2012) and job resources (Förster and Duchek, 2017), but can also actively promote the emotions and attitudes of employees (Pedersen and Jeppesen, 2012; Yucel, 2019), thereby positively affecting their resilience.

From the perspective of the health impairment process, SLP “transfers” traditionally managed tasks to each member (Hiller et al., 2006) and involves “sharing” multiple responsibilities among team members (Pearce and Sims, 2002). These additional leadership roles can result in role overload. Moreover, SLP leads to more in-depth and complex interpersonal relationships (Lau et al., 2021) through its requirements for guiding and helping team members, which could also strengthen the role overload in team members' relationships. Role overload is a typical job demand that could lead to more employee stress (Duxbury et al., 2018), emotional exhaustion (Ahmad, 2010), reduce employee work engagement (Deng et al., 2021), and promote more work interference with family life (Walumbwa et al., 2022), thus adversely affecting employee resilience (Chen et al., 2021; Sommer et al., 2016). Therefore, our perspective is that SLP could inadvertently undermine resilience among employees by escalating role overload.

Previous literature outlines the effect of SLP on employee outcomes is affected by boundary factors (Wu et al., 2020). Specifically, under a higher level of goal clarity can further affirm the association between SLP and job arrangement flexibility through helping individuals better guide managers' behaviors (Rizzo et al., 1970), better fulfill the leadership responsibilities assigned by SLP and makes a greater contribution to information sharing and teamwork (Anderson and West, 1996). Meanwhile, clearer organizational goals can not only help individuals better fulfill their leadership roles (Fürstenberg et al., 2021; Rizzo et al., 1970), but also support them in completing more out-of-role behaviors (Wright et al., 2012) and multitasking management (Patanakul et al., 2016). This can effectively alleviate the role pressure exerted on employees through SLP and promote employee resilience. Thus, we suggest that a higher level of goal clarity can reduce the influence of SLP on role overload.

In summary, we explored the dual impact of SLP on employee resilience and further examined the moderating role of goal clarity in the aforementioned relationship. The theoretical implications may follow three aspects. First, by exploring how SLP promotes employees' resilience through FWA, this study compensates for the lack of an interconnection between SLP practices and employee resilience (Liang et al., 2021), and further enriches our understanding of the impact of SLP on employees. Second, by investigating the research that SLP inhibits employee resilience through role overload, this work enhances our comprehensive understanding of the link between SLP and employee resilience, as well as our comprehension of the potential dark side of SLP. Third, by exploring the moderating effect of goal clarity on the double-edged interplay between SLP practices and employee resilience, we increase our understanding of the boundary utility of the mechanism affecting employee resilience.

## 2 Theoretical background and hypothesis development

### 2.1 The relationship between shared leadership and employee resilience

With regard to the relationship between SLP and employee resilience, prior scholarly endeavors have established a robust correlation between employees' emotional intelligence and their resilience (Förster and Duchek, 2017). Existing empirical evidence underscores the significant enhancement of employees' emotional intelligence by SLP (Zhang et al., 2024). Similarly, research has demonstrated that social support from both leaders and peers positively augments employee resilience (Kuntz et al., 2017). SLP, which is characterized by mutual support and care among team members (Nazarpoori, 2017), fosters a work environment conducive to such social support. Building on this rationale, we postulate that the relationship between SLP and employee resilience is positive. Additional studies further corroborate this perspective. For instance, previous research has shown that SLP actively fosters employee engagement in teamwork (Klasmeier and Rowold, 2022). Relevant research on employee resilience indicates that employee engagement, in turn, actively bolsters employee resilience (Ablett and Jones, 2007). Consequently, we formulate the following hypothesis:

*Hypothesis 1: The relationship between shared leadership and employee resilience is positive*.

### 2.2 Shared leadership's “bright side” on employee resilience

Flexible work arrangements are a typical job resource (Kelly et al., 2020; Yeves et al., 2022) that has been proven to be affected by leadership style (Jauhar and Suratman, 2022). Based on the JD-R model (Bakker and Demerouti, 2007; Bakker et al., 2003; Demerouti et al., 2001), we believe that SLP enhances the FWA of team employees, making it an important job resource that could trigger the motivational process of JD-R model (Schaufeli et al., 2009) and promote employee resilience.

We believe that SLP promotes employees' FWA. The specific reasons are as two follows. First, SLP endows employees with more leadership roles (Pearce and Conger, 2003), which not only leads to more active decision-making participation in arranging work (Muethel and Hoegl, 2011), but can also help improve their power and influence in teams and organizations (Azeem et al., 2021; DeRue, 2011; Sinha et al., 2021), which are indicated to be important promotion factors of FWA (Rousseau et al., 2006). SLP has transitioned from focusing on individual-level phenomena and leader-centric approaches to a dynamic, collaborative, and group-level perspective (Pearce, 2004). It emphasizes the collective exertion of leadership, which is perceived as a collection of roles that any team member can undertake (Shane Wood and Fields, 2007). In leadership work tasks, decision-making and allocation occupy a certain proportion, which largely determines the direction and distribution of team resources. Therefore, teams implementing SLP not only passively receive arrangements, but also actively participate in decision-making by arranging their own work (Muethel and Hoegl, 2011). Moreover, previous research indicated that employees who were of high status and highly valued are contributed to higher FWA (Rousseau et al., 2006), and an extra leader role could help improve their power and influence in teams and organizations (Azeem et al., 2021; DeRue, 2011; Sinha et al., 2021), allowing individuals to gain more access to a higher level of FWA.

Second, SLP places great emphasis on overall coordination and information sharing within a team (Hoch, 2014; Imam and Zaheer, 2021), and emphasizes mutual support and care among members (Nazarpoori, 2017), all of which promote FWA with more information resources and member supports. SLP motivates the workforce to partake in joint decision-making and support one another in the pursuit of common objectives (Carson et al., 2007). This culture of information sharing (Imam and Zaheer, 2021) and collaborative decision-making allows employees to be aware of specific work arrangements and enables employees to understand the capabilities and needs of other team members. This allows individuals to make work arrangements with more detailed and specific information resources and further enhances their FWA. What's more, the SLP atmosphere of assisting in completing work tasks allows for better communication among employees (Hiller et al., 2006) and helps members handle emergencies outside work, thus promoting individual FWA.

In summary, SLP empowers employees with higher decision-making power, emphasizes information flow to make employees aware of the team's capabilities and arrangements, and creates a harmonious atmosphere of mutual assistance within the team, all of which could provide employees with higher FWA. This study proposed that SLP promotes FWA among employees.

*Hypothesis 2: SLP promotes employees' FWA*.

Based on the JD–R model (Demerouti et al., 2001), we also believe that FWA motivated by a higher level of SLP could trigger the motivational process (Schaufeli et al., 2009) and make it benefit to employee resilience (Kuntz et al., 2017). The JD–R model proposes that the factors that individuals obtain in the workplace that are beneficial to their physical or psychological wellbeing can be classified as job resources (Demerouti et al., 2001). It serves as fuel to initiate motivational processes in individuals. By obtaining certain job resources, individuals feel that their health, development, and growth are guaranteed, which leads to greater engagement in subsequent work processes and positive work outcomes (Bauer et al., 2014).

Previous studies have shown that individual resilience is influenced by personal and job resources (Hartmann et al., 2020). Job resources include physiological, psychological, organizational, and social resources provided to individuals at work. FWA enables employees to arrange work independently, which can promote an internal locus of control under a higher level of FWA. Moreover, FWA can positively contribute to employee job satisfaction (Yucel, 2019) and mental health (Yeves et al., 2022). All of those make FWA is regarded as a typical job resource (Kelly et al., 2020; Yeves et al., 2022) and is indicated to promote employee work and organizational engagement (Pedersen and Jeppesen, 2012). This makes FWA which is stimulated by a higher level of SLP fit the motivational process of JD-R model that could lead more positive work outcomes (Bauer et al., 2014). More specially, previous research showed that internal control locus (Türk-Kurtça and Kocatürk, 2020), work engagement (Ablett and Jones, 2007), and positive emotions (Sommer et al., 2016) can promote employee resilience. In this logic, we believe that more job resources provided by higher FWA promote individuals' positive development (Bakker and Demerouti, 2007) and engagement in work processes (Pedersen and Jeppesen, 2012), which triggers the motivational process and make it benefit to employee resilience (Kuntz et al., 2017).

In summary, this study suggests that SLP promotes employee resilience by increasing employees' perceived FWA.

*Hypothesis 3: The promotion of FWA by SLP can positively affect employee resilience, thus FWA positively mediating the link between SLP and employees resilience*.

### 2.3 Shared leadership's “dark side” on employee resilience

Previous investigations have indicated that different leadership approaches can significantly impact the likelihood of role overload (Vullinghs et al., 2020). From the perspective of the JD-R model (Bakker and Demerouti, 2007; Bakker et al., 2003; Demerouti et al., 2001), we believed that SLP could also lead employees have more additional leadership roles and extra interpersonal responsibilities, all of those could increase role overload for employees, which could trigger the health impairment process of JD-R model (Schaufeli et al., 2009) and inhibit their resilience.

The specific reasons for how SLP could increase employee role overload are as two follows. On the one hand, sharing leadership assigns additional leadership roles and demands to employees, which increases their role overload. the adoption of a The approach of SLP empowers members to collectively wield influence, participate in decision-making, and undertake responsibilities typically designated to higher management in pursuit of team goals (Shane Wood and Fields, 2007). The additional roles of leaders include not only power, but also multiple responsibilities (Pearce and Sims, 2002). In teams implementing SLP, tasks such as setting work goals and improving work methods, which were traditionally managed by superiors, are “transferred” to each member (Hiller et al., 2006). From an individual perspective, this means taking on more leadership roles and fulfilling additional leadership responsibilities, which can, to some extent, create leadership role overload for individuals. The core idea of SLP is to allow leadership to be shared or “rotated” among team members, which requires active cooperation from employees (Shane Wood and Fields, 2007). Previous research has shown that frequent interaction and influence between multiple leaders can create an “arena” (Greer et al., 2018), and members may vie for their power territory in this “arena,” leading to conflicts (Greer et al., 2017). This struggle for power requires employees to exert additional mental effort, leading to the aggravation of leader role overload.

On the other hand, SLP also immerses employees in more in-depth and complex interpersonal relationships, increases the fulfillment of extra interpersonal responsibilities and requirement, and further aggravates their role overload. Compared with traditional leadership styles, SLP requires team members to provide guidance to each other at appropriate times, resulting in closer relationships between employees (Evans et al., 2021). This increased contact and assistance can make employees' interpersonal relationships more in-depth and complex (Lau et al., 2021). This may increase the work of the employee's interpersonal responsibilities, thus creating a role overload from the interpersonal relationship role. Moreover, SLP encourages a mutually supportive environment to allow investments in team members' leadership (Ali et al., 2020). It encourages employees to take responsibility for creating positive interpersonal relationships (DeRue and Ashford, 2010) and jointly fulfill organizational responsibilities (Wu et al., 2020), which leads them to take on more interpersonal responsibilities (Evans et al., 2021), resulting in more individual sacrifices and fulfilling responsibilities that colleagues have failed to fulfill (Sanner et al., 2022). This could further exacerbate the interpersonal role overload on employees. In summary, SLP, which advocates leadership “rotation,” assigns additional leadership roles and responsibilities to employees, and increases employees the fulfillment of extra interpersonal responsibilities may bring role overload to employees.

*Hypothesis 4: SLP increases employee role overload*.

Based on the JD-R model (Demerouti et al., 2001), we also believe that Role overload, as a consequence of SLP, which is regard as a classic job requirement variable (Duxbury and Halinski, 2014), could trigger the health impairment process of JD-R model (Schaufeli et al., 2009) and reduce employee resilience (Kuntz et al., 2017). JD–R model believes the continuous demands placed on individuals can be categorized as job demands, such as heavy workloads and role conflicts (Demerouti et al., 2001). The accumulation of these “negative factors” can lead individuals to develop defensive strategies (Chen and Qi, 2022; Hetty Van Emmerik et al., 2009), which can weaken their motivation to exert effort at work, reduce their proactivity, and adversely affect subsequent work outcomes (Demerouti et al., 2001).

Role overload refers to the pressure that employees experience owing to time constraints or difficulty in supporting the completion of various role tasks (Bolino and Turnley, 2005), which is a classic job requirement variable (Duxbury and Halinski, 2014). Role overload's excessive job requirements and scarce job resources could trigger health impairment process, which reveals that employees' energy will be constantly depleted in the excessive job requirements and scarce job resources, which may eventually lead to energy exhaustion then have a negative impact on employees (Schaufeli et al., 2009; Lewig et al., 2007). Specific to this study, the excessive role task requirements of role overload could lead to more employee stress (Duxbury et al., 2018) emotional exhaustion (Ahmad, 2010) and less work engagement (Deng et al., 2021). Too much stress could deplete individual physical and mental resources (Mui Hung Kee et al., 2024). Existing literature has identified stress as a significant inhibitor of employee resilience (Wu et al., 2013), proved the negative emotional states reduce employee resilience (Green et al., 2011) and also indicated that promoting effect of work engagement on employee resilience (Ablett and Jones, 2007), it is expected that role overload could further reduce employee resilience by trigger health impairment process.

Thus, based on the JD-R model (Demerouti et al., 2001), it is rationale to expect that SLP leads to more role overload among employees, thereby trigger the health impairment process (Schaufeli et al., 2009) and reducing their resilience. Thus, employee role overload mediates the negative effect of SLP on employee resilience.

*Hypothesis 5: Role overload, as a consequence of SLP, could reduce employee resilience, with role overload serving as a negative mediator in the SLP-employee resilience nexus*.

### 2.4 Moderating effect of goal clarity

Goal clarity refers to work outcomes, objectives, and purposes within an organization are explicitly stated and defined (Sawyer, 1992). Goal clarity is a type of work resource provided by organization (Fürstenberg et al., 2021). Goal clarity, as a core requirement for employees in a rapidly changing era (Stouten et al., 2018), can positively affect employees' decisions and outcomes.

Specifically, we believed that goal clarity positively moderated the link between SLP and FWA, further promoting employee resilience. First, SLP assigns employees more leadership roles, and we believe that under a higher goal clarity further strengthens employees' leadership role power and influence, empowering employees higher FWA and resilience. Goal clarity positively affects individual work enthusiasm (Perry and Porter, 1982). Goals influence performance by guiding focus, stimulating effort, increasing persistence, and fostering strategic development (Locke et al., 1981). Due to the greater management power and influence bestowed upon employees by SLP, it promotes employees flexibility in work arrangements (Rousseau et al., 2006). Existing research shows that the existence of clear behavioral requirements in an organization are of great help in guiding managers' behavior (Rizzo et al., 1970). Under higher goal clarity, not only will subordinates' work autonomy and engagement significantly improve (Fürstenberg et al., 2021), but team performance will also be greatly enhanced (Sun et al., 2014). Therefore, the influence of SLP on employee leadership roles is further strengthened under higher goal clarity.

Second, existing research suggests that higher goal clarity lead to better performance (Heine et al., 2023; Sonnentag and Volmer, 2010), indicating the correlation between higher goal clarity and higher effectiveness and efficiency (Van der Hoek et al., 2018). Since higher goal clarity not only includes clearer information about individual work goals and responsibilities, but also includes effective information about the process of how individuals complete work goals and responsibility (Sawyer, 1992), employees will receive clearer and more effective work information under higher goal clarity, which helps them better arrange work based on those effective information. Conversely, lower goal clarity may lead employees to face conflicting information (Heine et al., 2023), this will bring confusion to employee information processing and greatly reduce the SLP's benefit to FWA through information sharing. What's more, existing research shows that with higher goal clarity, the team's collaboration function and open communication will be further strengthened (Saavedra et al., 1993), so with clearer goals, the team cooperation advocated by SLP will be further strengthened. As such, we proposed the following hypothesis:

*Hypothesis 6: The presence of goal clarity intensifies the beneficial link between SLP and FWA. As goal clarity escalates, so does the strength of this positive interconnection*.

We also believe that goal clarity negatively moderated the positive link between SLP and role overload. First, in terms of the role overload brought by additional leadership roles to employees, under a higher goal clarity can not only better guide leadership behavior (Frese et al., 1997), but also actively promote the work autonomy and work engagement of subordinates (Fürstenberg et al., 2021) and promote team performance (Lui et al., 2023), all of which could help employees better complete the tasks of leadership roles. This greatly reduces the role overload associated with additional leadership roles, and further enhances employee resilience.

Second, higher goal clarity represents members' clearer understanding and mutual recognition of organizational goals (Perry and Porter, 1982), as well as individuals' clearer cognition of the relationship between individual goals and overall goals (Sawyer, 1992), which will greatly reduce the negative impact caused by individuals' competition for power. At the same time, it will further clarify the goal boundaries among team members, separate individuals from the complex interpersonal network of colleagues, and reduce the impact of the extra interpersonal responsibilities from SLP on individual resilience.

Existing research also shows that goal clarity can not only help employees stay focused at work (Quinn, 2005), but it can also increase their work engagement (Whitaker et al., 2007), and higher goal clarity will actively promote employees' extra-role behavior (Wright et al., 2012) and support their multi-project management (Patanakul et al., 2016). All these results show that higher goal clarity will not only help individuals reduce the pressure brought about by role overload, but also help them perform better in multi-role tasks, thus reducing the negative effect of extra role responsibilities assigned by SLP.

*Hypothesis 7: Goal clarity negatively moderates the adversarial interaction between SLP and role overload. As clarity regarding objectives escalates, the unfavorable link between these elements diminishes*.

### 2.5 An integrative moderated mediation model

**Figure 1:**
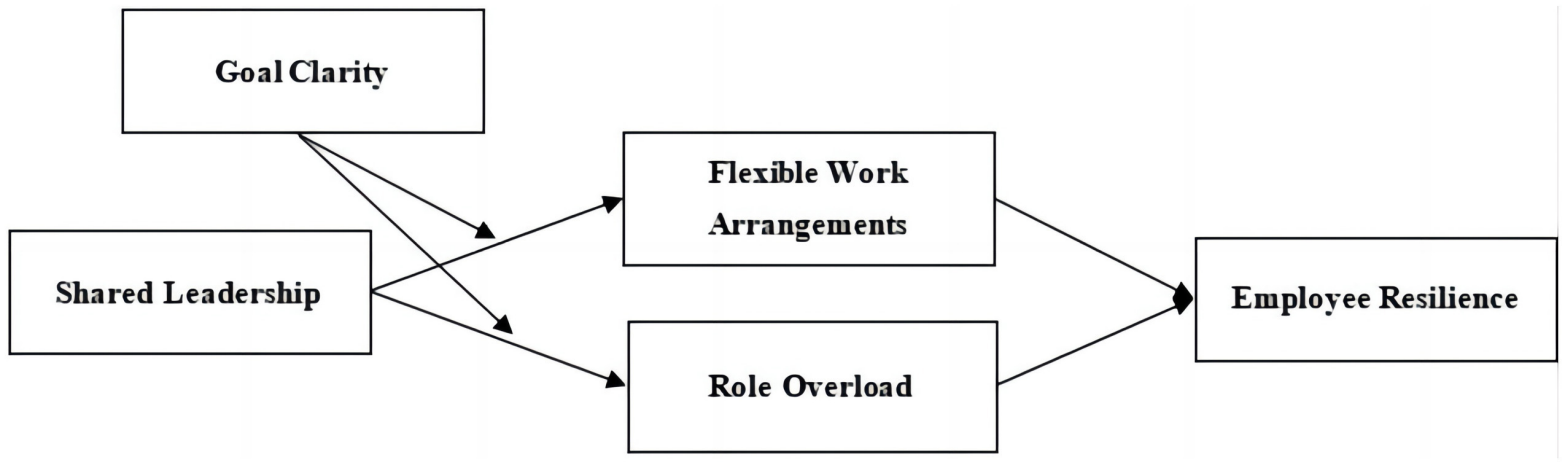
Overview of the hypothesized moderated mediation model.

Building upon the aforementioned hypotheses, we propose an integrated moderated mediation model (See Figure 1). The increased levels of SLP are expected to enhance employees' FWA, consequently benefiting their resilience. However, simultaneously, SLP may also lead to higher levels of role overload, which can negatively impact employee resilience. In light of this, the presence of high goal clarity can guide managers' behavior (Rizzo et al., 1970), improve performance (Gonzalez-Mulé et al., 2016), and support multi-project management (Patanakul et al., 2016), and could further strengthened the positive link between SLP and FWA. Goal clarity will also positively moderate the positive effect of SLP on employees' role overload, subsequently enhancing employee resilience. Consequently, we propose the following integrated moderated mediation model:

*Hypothesis 8: SLP have positive effects on employee resilience through increased FWA, while SLP also have potential negative effects on employee resilience through increased role overload. The first link of this mediation process will be moderated by goal clarity, such that under conditions of high goal clarity, the positive indirect effects will be stronger, and the negative indirect effect of SLP on role overload will be mitigated. These combined effects ultimately contribute to improved employee resilience*.

## 3 Methodology

### 3.1 Participants and procedure

First, drawing upon prior research on SLP (Chiu et al., 2016; Lyndon et al., 2020), we confirmed that the selected companies and their members met the following four criteria: (1) Participants' work content must address diverse customer needs; (2) Participants' work content is interdependent among team members; (3) Participants can assume leadership roles in various areas based on work dimensions; and (4) Participants have a leader responsible for team performance (Chiu et al., 2016; Lyndon et al., 2020).

Second, according to the above requirements, to ensure the randomness and representativeness of the sample, qualified convenience sampling samples were selected at the whole mainland of China, and finally eight companies were selected registered in Beijing, Shanghai, Hunan, Chongqing, Sichuan, Zhejiang, Guangdong, and Fujian. Five of the eight selected companies are state-owned enterprises and the remaining three are private enterprises. These companies encompassed eight industries, including internet, real estate, education and training, clothing manufacturing, finance, machinery manufacturing, food processing, and others.

Third, we communicated with company senior management regarding the purpose and significance of this research, garnering their support to ensure the seamless progression of the project. Subsequently, we collaborated with each company's human resource managers to randomly select participants who met the aforementioned criteria. Participants were sourced from administrative management, human resources management, training and education, project, and consulting departments. Notably, our survey target primarily focused on employees rather than teams.

Fourth, surveys were electronically disseminated through the reputable third-party survey platform Sojump, which facilitated sample recruitment from its online user base and through referrals. Incentives were provided to enhance participant engagement. After determining the number of participants and their respective enterprise information. To ensure anonymity and minimize potential common method biases, we collected the last four digits of each subject's mobile phone number as its unique code in the study to facilitate data collection over two distinct intervals. We confirmed the written informed consent of each subject to participate in this study through the contact information of the participants. The research team logged into the Sojump online questionnaire collection platform to prepare and conduct a trial run of the questionnaire at both time points. Sojump has been accepted and recognized by top international journals, such as the Academy of Management Journal (Zhang et al., 2023) and the Journal of Applied Psychology (Ferris et al., 2018).

The overall distribution was finalized after confirmation by the research team. At the first time point, we collected evaluations from individual employees on SLP, goal clarity, and control variables (including employee age, gender, and education level). A total of 504 questionnaires were sent out, with 402 collected at the first time point (response rate = 79.7%).

Fifth, after 2 weeks later, a second time-point questionnaire was distributed to the 402 employees who participated in the first time-point survey, measuring their perceived role overload, family-work conflict (FWC), and employee resilience. A total of 246 questionnaires were collected at the second time point (response rate = 61.2%). The participants comprised employees aged 22–55, with a mean age of 32.25 years (SD = 7.87). Among them, 35.3% held non-management positions, 43.9% were in front-line management positions, 58.1% had a bachelor's degree, and 23.5% had a graduate degree or higher.

### 3.2 Measurements

The items of the questionnaire scales were all comes from established scales in international top journals with high content validity and reliability. Since the scales are all in English, while we conducted the research in the Chinese context, we used the conventional method of translation-back translation (Brislin, 1986) to ensure accuracy of the measures. The English scales were translated into Chinese by two bilingual Ph.D. students and then were translated back into English by two other bilingual Ph.D. students. The four students discussed and resolved the discrepancies and finalized the Chinese scales. Employee resilience, FWA and goal clarity rated on a 5-point Likert scale, where responses range from 1 (strongly disagree) to 5 (strongly agree), while SLP and role overload employed a 7-point Likert scale, extending from 1 (highly inconsistent) to 7 (highly consistent). The Cronbach's alpha of SLP, FWA, employee resilience, role overload, and goal clarity is 0.959, 0.823, 0.887, 0.837, and 0.868. At the same time, we further explain the number, source and sample questions items contained in these scales.

#### 3.2.1 Shared leadership

The assessment of SLP was conducted through a multifaceted 25-item instrument (Hiller et al., 2006). An illustrative item is “Team members Diagnosing problems quickly.”

#### 3.2.2 Employee resilience

The construct of employee resilience was evaluated using a 9-item instrument established by Caniëls and Hatak (2022). One question items, for example, is “I bear a heavy workload without getting discouraged.”

#### 3.2.3 Flexible work arrangements

The measure of work arrangement flexibility employed a unidimensional 4-item scale, designed by Piszczek et al. (2021). A sample item is “I have input into making my schedule.”

#### 3.2.4 Role overload

Role overload was determined using an 8-item, single dimension scale formulated by Jensen et al. (2013). A sample item is “I have unrealistic time pressures.”

#### 3.2.5 Goal clarity

The clarity of organizational goals was quantified utilizing a 5-item, one-dimensional scale devised by Sawyer (1992). An illustrative example is “I know clearly about the expected results of my work.”

#### 3.2.6 Control variables

In light of the various studies implying gender (Bridges et al., 2023; Huang et al., 2019) could significantly affect employee resilience, and age (Scheibe et al., 2022) education level (Ang et al., 2018) have a positive effect on employee resilience. The current research refer to Pink-Harper and Rauhaus' research on the antecedents of employee resilience (Pink-Harper and Rauhaus, 2017), and accounted for gender, age, level of education, and position as control variables.

## 4 Results

### 4.1 Common method variance

Regarding the statistical method employed, the current investigation utilized the SPSS25.0 for executing a Harman's single-factor test to assess potential common method bias within the measurement instruments. Subsequently, confirmatory factor analysis (CFA) was conducted using AMOS24.0 to ascertain the construct validity of the instruments. Following previous leader style empirical studies (Mui Hung Kee et al., 2020), descriptive statistics, assessments of inter-variable correlations were also carried out via SPSS25.0. To probe into the mediating, moderating effects and conditional indirect effects posited in our hypothesis, hierarchical regression analyses by PROCESS macro for SPSS (model 4 for hypothesis 1–4 and model 7 for hypothesis 5–7), along with Bootstrap procedures (reiterations set at 5,000 samples) were conducted.

Utilizing AMOS24.0, a confirmatory factor analysis was undertaken on variables including SLP, employee resilience, FWA, role overload, and goal clarity in Table 1. As see in Table 1 the hypothesized five-factor model exhibited the most robust fit (χ^2^*/df* = 1.127, *CFI* = 0.989, *TLI* = 0.988, *IFI* = 0.990, *RMSEA* = 0.021), outperforming other models including one-factor model (Δχ^2^ = 2429.256, Δ*df* = 10, *p* < 0.001), and demonstrating sound discriminant validity for each factor examined.

**Table 1:** Confirmatory factor analysis result.

**Model**	**χ^2^**	***df***	**χ^2^/*df***	**CFI**	**TLI**	**IFI**	**RMSEA**
One factor (SLP+GC+ FWA+ROV+ER)	2855.523	405	7.051	0.325	0.275	0.330	0.157
Two factors (SLP+GC+ FWA+ROV; ER)	1945.611	404	4.816	0.575	0.543	0.579	0.125
Three factors (SLP+GC+FWA; ROV; ER)	1348.190	402	3.354	0.739	0.718	0.742	0.098
Four factors (SLP+GC; FWA; ROV; ER)	1001.452	399	2.510	0.834	0.819	0.836	0.079
Five factors (SLP; GC; FWA; ROV; ER)	426.267	395	1.079	0.991	0.991	0.991	0.018

SLP, shared leadership; GC, goal clarity; FWA, flexible work arrangements; ROV, role overload; ER, employees resilience.

This research relied on self-reported data, which may introduce biases such as social desirability bias or recall bias. To mitigate these potential biases, we employed anonymous survey methods, assigning each participant a unique identifier across the two time points to ensure anonymity and match responses over time. Furthermore, we utilized scales with high reliability and validity from top journals to further reduce potential common method biases arising from self-reporting. Previous research indicated that the common method bias is not a major concern if the result of Harman single-factor test were below 40% (Podsakoff et al., 2003). This study's Harman single-factor test indicated that the cumulative variance explanation of the first factor in this research is 27.23%, below the rule of thumb threshold of 40%, indicated that common method bias is not a major concern in this study.

### 4.2 Descriptive statistics

As shown in Table 2, SLP is significantly positively correlated with FWA (*r* = 0.312, *p* < 0.001); and FWA is positive related to employee resilience (*r* = 0.457, *p* < 0.001). Furthermore, SLP is significantly positively correlated with role overload (*r* = 0.186, *p* < 0.01); and role overload is negative correlated with employees resilience (*r* = −0.131, *p* < 0.05). The above results provide preliminary data support for the hypothesis 1–4 of this study.

**Table 2:** Means, standard deviations, and correlations between variables.

**Variables**	**Mean**	**SD**	**1**	**2**	**3**	**4**	**5**	**6**	**7**	**8**	**9**
1	1.200	0.403	1								
2	3.050	0.646	−0.198^**^	1							
3	1.960	0.945	0.191^**^	−0.137^*^	1						
4	32.250	7.869	0.28^***^	−0.202^**^	0.700^***^	1					
5	4.198	0.920	0.076	−0.074	−0.026	−0.045	1				
6	3.683	0.870	0.115	−0.184^**^	−0.055	0.035	0.151^*^	1			
7	3.387	0.939	0.031	0.006	0.047	−0.025	0.312^***^	0.050	1		
8	4.272	0.853	0.124	−0.017	0.015	−0.014	0.186^**^	0.050	0.044	1	
9	3.353	0.760	−0.047	−0.016	−0.077	−0.062	0.277^***^	0.089	0.457^***^	−0.131^*^	1

N , 246; 1 , Gender; 2 , Education level; 3 , position; 4 , Age; 5 , shared leadership; 6 , goal clarity; 7 , flexible work arrangements; 8 , role overload; 9 , employees resilience significant at ^*^p < 0.05; ^**^p < 0.01; ^***^p < 0.001.

### 4.3 Hypothesis testing

Based on the method of Preacher et al. (2007), we use the PROCESS macro of Hayes (2012) to test this mediation mode. The SPSS PROCESS macro is widely used to test moderating and mediating effects, and the hypotheses of this study constitute a mediating model (Hayes, 2012). Specifically, this study uses PROCESS Model 4 to test and analyze the mediating effect, and PROCESS Model 7 to test and analyze the moderating effect. Five thousand iterations were used to generate bootstrap-based 95% *CI* (confidence intervals) and bias correction for indirect effects. The outcomes of our regression analysis confirm that the direct effect of SLP on employee resilience is indeed positive, with an effect size of 0.149 (*B*_*simple*_ = 0.149, *p* < 0.01, *95% CI* = [0.052, 0.246]). The results support our hypothesis 1 that the relationship between SLP and employee resilience is positive. Hypothesis 2 proposed that SLP have a positive effect on FWA. As shown in Table 3, results of model 2 indicate that SLP positively contribute to a higher level of FWA (Model 2, *B*_*simple*_ = 0.318, *p* < 0.001, *95% CI* = [0.195, 0.442]), supported hypothesis 2. Model 3 showed that FWA is positive related to employees resilience (Model 3, *B*_*simple*_ = 0.341, *p* < 0.001, *95% CI* = [0.246, 0.435]), and the positive mediating effect of FWA on the link between SLP and employee resilience was also confirmed (indirect effect = 0.108, *95% CI* = [0.061, 0.161]), supported hypothesis 3.

**Table 3:** Regression results for main, mediation effects (model 1–model 5).

**Variable**	**ER**		**FWA**		**ER**		**ROV**		**ER**	
	**Model 1**		**Model 2**		**Model 3**		**Model 4**		**Model 5**	
	***B***	***SE***	***B***	***SE***	***B***	***SE***	***B***	***SE***	***B***	***SE***
Gender	−0.116	0.123	0.039	0.150	−0.129	0.112	0.262	0.141	−0.089	0.111
Age	−0.017	0.075	0.044	0.092	−0.032	0.069	0.017	0.086	−0.029	0.067
Education	−0.056	0.070	0.123	0.085	−0.098	0.064	0.044	0.080	−0.091	0.063
Position	0.001	0.009	−0.012	0.011	0.039	0.008	−0.008	0.010	0.004	0.008
SLP	0.231^***^	0.051	0.318^***^	0.063	0.122^*^	0.049	0.163^**^	0.059	0.149^**^	0.049
FWA					0.341^***^	0.048			0.338^***^	0.047
ROV									−0.156^**^	0.051
*R^2^*	0.085		0.106		0.243		0.050		0.272	
*F*	4.4561^***^		5.7086^***^		12.7893^***^		2.5080^*^		12.7123^***^	

N, 246 Significant at: ^*^p < 0.05; ^**^p < 0.01; ^***^p < 0.001. SLP, shared leadership; GC, goal clarity; FWA, flexible work arrangements; ROV, role overload; ER, employees resilience.

Hypothesis 4 believed that SLP could lead employees have more overload. Results of model 4 indicate that SLP is positively related to role overload when added role overload as another mediator (Model 4, *B*_*simple*_ = 0.163, *p* < 0.01, *95% CI* = [0.047, 0.279]), supported hypothesis 4. Model 5 showed that role overload is negative related to employees resilience (Model 5, *B*_*simple*_ = −0.156, *p* < 0.01, *95% CI* = [−0.256, −0.056]), and the negative mediating effect of role overload on the link between SLP and employee resilience was also confirmed (indirect effect = −0.026, *95% CI* = [−0.054, −0.006]), supported hypothesis 5.

**Figure 2:**
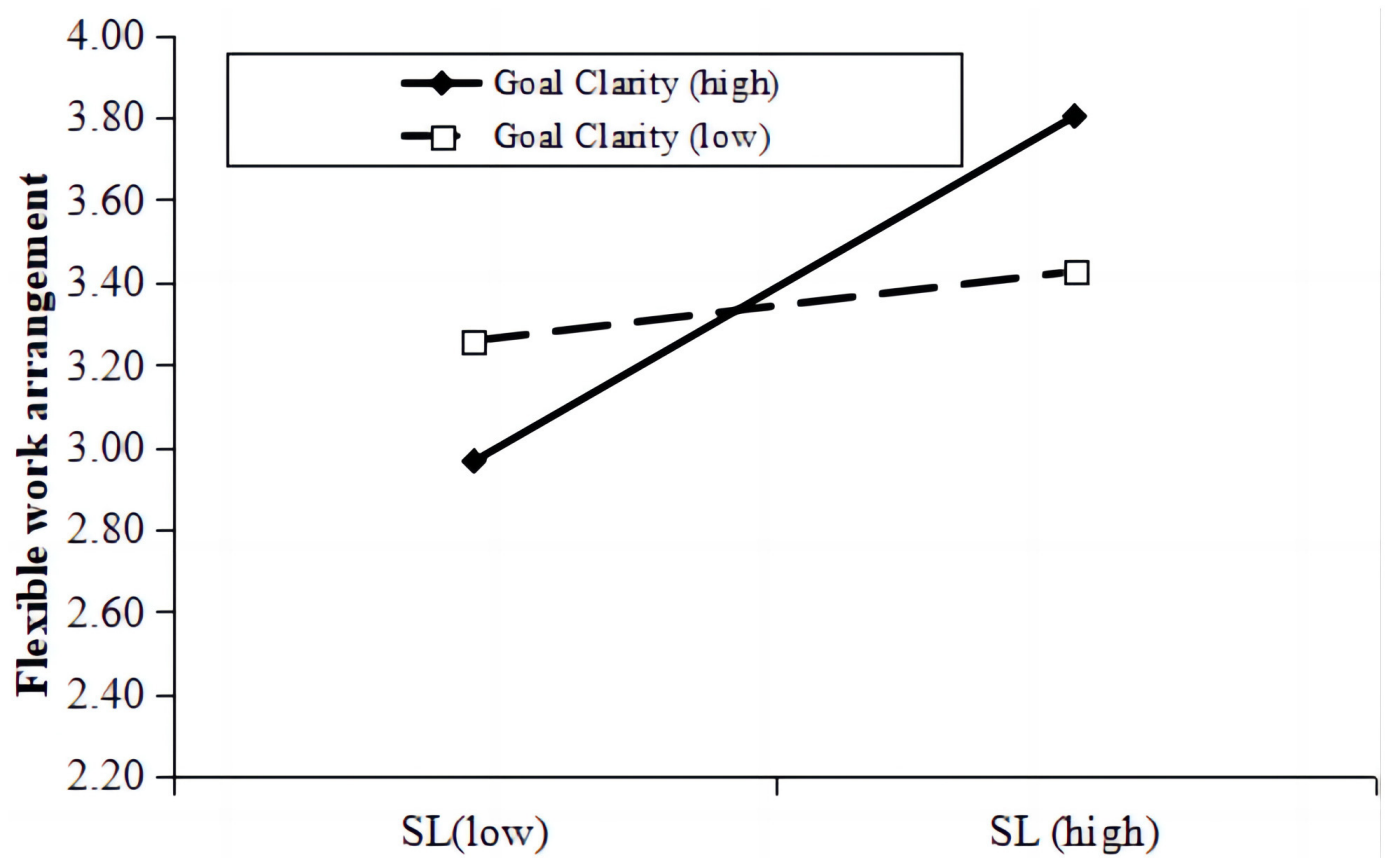
Moderating effect of goal clarity on the relationship between SLP and flexible work arrangement.

This study investigated the conditional influence of goal clarity on the dynamics between SLP and employee resilience. As shown in Table 4, the analysis unveiled that a fusion of SLP and high goal clarity markedly enhanced FWA, denoted by a significant moderation effect (*moderation effect* = 0.209, Model 6, *B*_*simple*_ = 0.273, *p* < 0.001, *95% CI* = [0.144, 0.402]), suggesting that a higher level of goal clarity amplifies the beneficial link between SLP and FWA. As depicted in Figure 2, the facilitative impact of SLP on FWA was notably pronounced at an elevated level of goal clarity (1 SD above the mean, *B*_*simple*_ = 0.086, *95% CI* = [0.286, 0.625], *p* < 0.001), yet this association did not hold true when goal clarity was diminished (1 SD below the mean, *B*_*simple*_ = 0.114, *95% CI* = [−0.133, 0.316], *p* = 0.423). Hence, Hypothesis 6 garnered empirical support.

**Table 4:** Regression results for moderation and mediation effects (model 5–model 8).

**Variable**	**FWA**		**ER**		**ROV**		**ER**	
	**Model 6**		**Model 7**		**Model 8**		**Model 9**	
	***B***	***SE***	***B***	***SE***	***B***	***SE***	***B***	***SE***
Gender	0.054	0.089	−0.129	0.112	0.240	0.140	−0.089	0.111
Age	0.046	0.150	−0.032	0.069	0.023	0.086	−0.029	0.067
Education	0.115	0.092	−0.098	0.064	0.057	0.079	−0.091	0.063
Position	−0.012	0.085	0.005	0.008	−0.008	0.010	0.004	0.008
SLP	0.273^***^	0.065	0.122^*^	0.049	0.205^***^	0.061	0.148^**^	0.049
FWA			0.341^***^	0.048			0.338^***^	0.047
ROV							−0.156^***^	0.051
GC	0.024	0.068			0.012	0.063		
SLP^*^GC	0.209^*^	0.088			−0.217^**^	0.083		
*R^2^*	0.127		0.243		0.077		0.272	
*F*	4.9513^***^		12.7893^***^		2.8296^**^		12.7123^**^	

N, 246 Significant at: ^*^p < 0.05; ^**^p < 0.01; ^***^p < 0.001. SLP, shared leadership; GC, goal clarity; FWA, flexible work arrangements; ROV, role overload; ER, employees resilience.

Hypothesis 7 posited a negative moderation by goal clarity on the link between SLP and role overload. The interaction between SLP and goal clarity was found to significantly reduce role overload (*moderation effect* = −0.217, Model 8, *B*_*simple*_ = 0.205, *p* < 0.001, *95% CI* = [−0.380, −0.055]). As depicted in Figure 2, the impact of SLP on role overload was not pronounced at an elevated level of goal clarity (1 SD above the mean, *B*_*simple*_ = 0.165, *95% CI* = [−0.142, 0.175]), yet this association was strengthen when goal clarity was diminished (1 SD below the mean, *B*_*simple*_ = 0.394, *95% CI* = [0.185, 0.604], *p* < 0.001), supported hypothesis 7.

**Figure 3:**
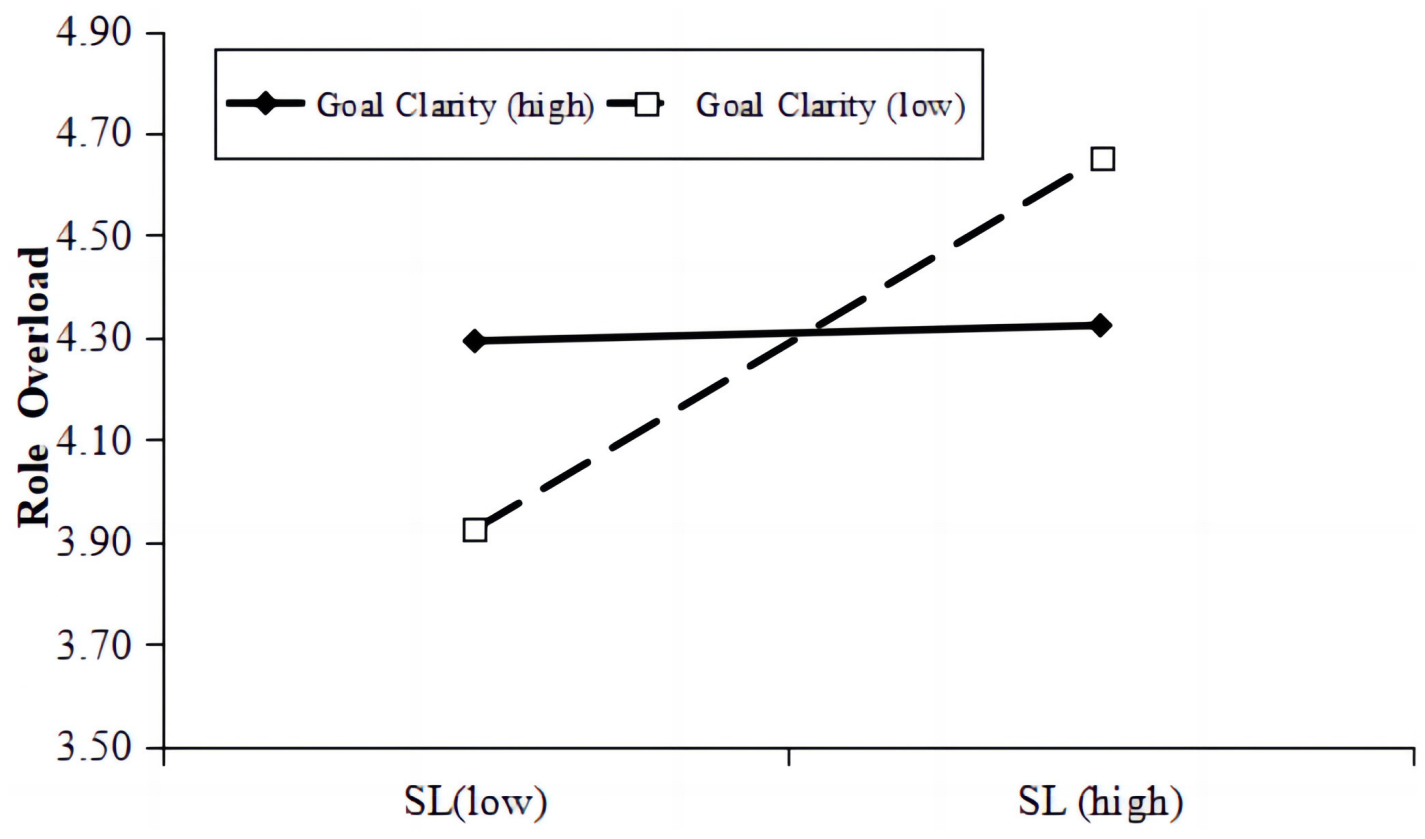
Moderating effect of goal clarity on the relationship between SLP and role overload.

As shown in Figure 2, when under a high level of goal clarity, the result showed a significantly positive conditional indirect effect of SLP on employees resilience through FWA (1 SD above the mean, *indirect effect* = 0.155, *95% CI* = [0.092, 0.226]), however it was not significantly at a lower level of goal clarity t (1 SD below the mean, *indirect effect* = 0.031, *95% CI* = [−0.042, 0.109]). As shown in Figure 3, the moderated mediation index of the FWA was 0.071 (*95% CI* = [0.017, 0.128]). Meanwhile, the conditional indirect effect of role overload was negatively and significant in the full moderated mediation model under a lower level of goal clarity (1 SD below the mean, *indirect effect* = −0.062, *95% CI* = [−0.11, −0.022]), whereas it was not significant under a higher level of goal clarity (1 SD above the mean, *indirect effect* = −0.03, *95% CI* = [−0.032, 0.022]). The moderated mediation index of role overload was 0.034 (*95% CI* = [0.008, 0.065]). Hypothesis 8 was supported.

## 5 Discussion

### 5.1 General discussion

This investigation centers on employee resilience, constructing a moderated dual-edged model under the framework of the Job Demands-Resources Model to explore the dual-edged influence of SLP on employee resilience and its boundary conditions. The findings indicate that the relationship between SLP and employee resilience is positive. Furthermore, SLP's bright side, that is, its enhancement of employee resilience through increased FWA, substantiates Hypotheses 2 and 3. These results align with existing research on the positive effects of SLP at the employee level (e.g., Hoch, 2013; Fu et al., 2020), and made up for the insufficient research on SLP to the individual capability (Liang et al., 2021). Conversely, the potential dark side of SLP was examined, revealing that it might increase employees' role overload, consequently diminishing their resilience, supporting Hypotheses 4 and 5. This echoed the dark side of SLP research that SLP may lead to reduce individual creativity (Wang and Peng, 2022) and team performance (Han et al., 2021), and further expanded on the dark side of SLP for employee resilience. Moreover, the study finding that organizational goal clarity not only positively moderates the effect of SLP on promoting FWA but also mitigates its impact on employee role overload, confirming Hypotheses 6–8. This further enriches previous research on the boundary factors of employee resilience (e.g., Kuntz et al., 2017; Varshney, 2022; Welbourne et al., 2015; Zhou and Zheng, 2022) and SLP (e.g., Liu et al., 2022). As a pivotal work resource, organizational goal clarity not only fosters leadership capabilities in employees, significantly enhancing subordinate engagement (Fürstenberg et al., 2021), but also envisions a greater contribution to information sharing and teamwork (Anderson and West, 1996). This can further assist employees in fulfilling leadership responsibilities delegated by SLP, augmenting their influence and reinforcing the positive effect of SLP on FWA (Rousseau et al., 2006) and employee resilience. Furthermore, under heightened organizational goal clarity, it also supports employees in executing additional leadership roles assigned by SLP more effectively, engaging in more out-of-role behaviors (Wright et al., 2012), and managing multitasking (Patanakul et al., 2016). This can attenuate the negative impact of SLP on employee role overload, thereby suppressing its adverse influence on employee resilience.

### 5.2 Theoretical contributions

First, this research explored the link between SLP and employees resilience. This contribute to our understanding of SLP's effects on employees work capability, and broaden the scope of inquiry into the antecedents of employee resilience. Previous research indicated that little attention have paid to how SLP affect employees capability (Liang et al., 2021), and scholars' call for further exploration the link between SLP and wellbeing (Zhu et al., 2018). This research responded to those research calls, enriches the exploration of the SLP's after-effects on employees capability. Moreover, antecedent variables on employee resilience generally focuses on individually centered leadership, such as narcissistic leadership (Li and Tong, 2021), sustainable leadership (Iqbal and Piwowar-Sulej, 2022), and authentic leadership (Mao et al., 2022), while neglecting the SLP styles. In addition, the existing research on SLP and resilience explored the impact of SLP on organizational resilience (Gichuhi, 2021) and team resilience (Salas-Vallina et al., 2022). The current lack of in-depth investigation of shared relationships with employee resilience was also addressed in this study. Therefore, this study also broadens research on the leadership antecedents of employee resilience.

Second, the negative effects of SLP on employee resilience was explored. Such insights further enrich our nuanced comprehension of SLP's repercussions on employee-related consequences. While there is some controversy in prior studies examining how SLP may alleviate job role conflict and reduce role ambiguity (Shane Wood and Fields, 2007), as well as role conflict among employees (Wang and Peng, 2022). According to our empirical results, shared roles may lead to greater employee role overload and reduce employee resilience. This further enhances our understanding of the antecedents that inhibit employee resilience. Significantly, most of previous research focus on the “bright side” of SLP, for example, SLP could improved organizational performance (Cooper et al., 2014), team innovation (Hoch, 2013; Liang et al., 2021), team performance (Siangchokyoo and Klinger, 2022), team creativity (Ali et al., 2020), fair reward (Grille et al., 2015), employee innovative behavior (Hoch, 2013), proactive behaviors (Fu et al., 2020), and so on. While only a few studies focusing on the “dark side” (e.g., Wang and Peng, 2022). Scholars may have exaggerated the benefits of SLP while ignoring its costs (Zhu et al., 2018). Previous studies indicate this leadership may not be as perfect as imagined (Wang and Peng, 2022). This study integrates the perspectives of reinforcement and weakening effects and believes that SLP not only increases employee resilience by increasing their FWA, but may also make employees perceive role overload and reduce their resilience. This helps to adopt a balanced approach to the potential risks and advantages of SLP, and a more holistic comprehension of its correlation with employee resilience can be attained.

Third, by assessing the moderating effect of goal, we not only extends the SLP practical relevance, but also deepens insights into the moderating factors influencing employees resilience. Previous moderating role in this filed focus on the individual or team level, such as opportunity recognition (Yu et al., 2022) and innovative self-efficacy (Liu et al., 2022). By introducing goal clarity as a moderating factor, our study not only uncovers the organizational factors that can optimize the effectiveness of SLP, but also find the boundary factors that reduce the potential negative impact of sharing type. This study also contributes to research on employee resilience. Most previous research on the role of boundaries affecting individual resilience has focused on ethnicity (Welbourne et al., 2015), work adaptability (Zhou and Zheng, 2022), machiavellianism (Varshney, 2022), promotion and prevention focus (Kuntz et al., 2017), and so on. This research not only offers fresh boundaries condition at organizational level, but also enriches our understanding of the boundary factors that benefit to employee resilience.

### 5.3 Managerial implications

First, our research indicates that SLP enhances employee resilience by promoting FWA. This may provide HR managers who want to improve employees' resilience with new practice ideas from the perspective of leadership style. On the one hand, Policy-makers in organizations can incorporate guidelines that encourage leadership styles that promote higher levels of SLP, such as by further increasing employees' job security. On the other hand, existing research shows that companies can increase the flexibility of employees' work arrangements through highly professional “I-Deals” terms in employee relationship (Kelly et al., 2020) and emotional intelligence (Lee et al., 2011), thus policies can advocate for the professionalization of “I-Deals” and emotional intelligence training programs to further enhance employee resilience.

Second, we found that SLP could intensifies employee's role overload and further reduce employee resilience. This finding may offer some early warnings for organizations that are fully implementing SLP practices, with the aim of mitigating the negative impacts on employee resilience during the implementation process. Specifically, the role overload status of employees when conducting SLP should be appreciated. Previous research indicated that higher perceived organization support (Zhang et al., 2022) and leader members exchange (Tang and Vandenberghe, 2021) could reduce the negative e of role overload on employees, policies that promote higher perceived organizational support and leader-member exchange (LMX) can be instrumental in buffering the negative effects of role overload.

Third, the goal clarity can enhance the positive sharing effect on the FWA, and further enhances employees resilience, the findings of this study provide valuable insights for managers on how to further amplify the positive effects of SLP on employee resilience in practical management scenarios. Previous studies have shown that companies can better promote process clarity and further enhance goal clarity by implementing more directive behavior and participative leader behavior (Sawyer, 1992). Therefore, by promoting clarity in goals and processes, managers can enhance the effectiveness of SLP. Implementing directive and participative leadership behaviors can help in achieving this clarity. For instance, setting clear, achievable targets, and involving employees in decision-making processes can foster a sense of purpose and direction, thereby enhancing employee resilience.

## 6 Limitations and directions for future research

This research's data were collected through a relatively simple two-point-time questionnaire survey, future studies could benefit from incorporating a longitudinal approach or experimental study, which would provide a more robust analysis of cause-and-effect relationships and allow for a better understanding of the long-term impacts of the SLP on employee resilience, capturing the evolving dynamics of the workplace and potentially revealing delayed effects not apparent in shorter-term studies.

Even though we utilized G^*^power software to calculate the minimum sample size required for correlation analysis and linear regression, and indicating that the sample size for studies are statistically appropriate, our sample size of 264 participants across two time points may be still relatively small compared to some large sample exemplary studies. Future research could replicate our findings with a larger sample size to enhance the ability to generalize the results to a broader population. Furthermore, although our Harman single-factor test indicated that that common method bias is not a major concern in this research, we encourage the future research adopt other-evaluation multi-source methods for investigation to further reduce common method biases resulting from self-reports.

Furthermore, although based previous employee resilience research (Ang et al., 2018; Bridges et al., 2023; Huang et al., 2019; Scheibe et al., 2022) and refer to Pink-Harper and Rauhaus' research on the antecedents of employee resilience (Pink-Harper and Rauhaus, 2017), we accounted for gender, age, level of education and position as control variables, we still encourage the inclusion of more control variables in the future, such as socioeconomic status, to improve the study's accuracy and make the findings more robust.

Finally, our research focuses on a specific organizational context, which provides deep insights but also limits the generalizability of the conclusions. For example, the aftereffects of SLP will show different results under different levels of culture context (Erkutlu, 2012), and considering the impact of culture factors on employee resilience (Sulphey, 2020), expanding the study to include cultural settings, diverse industries and organizational sizes could enrich the understanding of SLP's effects across various contexts. This would also address how different environmental and organizational cultures influence the relationship between SLP and employee resilience and enhance the generalizability of the research's conclusions.

## 7 Conclusion

The current study aims to dissect the dual-edged effects and underlying mechanisms of SLP on employee resilience. Within the scaffolding of the Job Demands-Resources Model (Bakker and Demerouti, 2007; Bakker et al., 2003; Demerouti et al., 2001), Firstly, the results of the empirical study support our hypothesis that the relationship between SLP and employee resilience is positive. Secondly, not only does the research validate the positive influence of SLP on employee resilience from the perspective of the motivational process (Schaufeli et al., 2009), but it also confirms from the health impairment process (Schaufeli et al., 2009) that SLP may inadvertently escalate employee role overload, thus eroding resilience. Thirdly, our study demonstrates that organizational goal clarity can further amplify the salutary role of SLP in promoting FWA, thereby elevating employee resilience, while simultaneously reducing the potential resilience depletion due to role overload induced by SLP. This provides a basis for magnifying the positive aspects of SLP on employee resilience and, more crucially, offers effective strategies to counteract the dark side of SLP on employee resilience. It assists employees in coping effectively with the potential role overload, that SLP may engender. By bolstering individual resilience, employees can more positively navigate work and family environments, enhancing their wellbeing. This research not only aids in a more comprehensive understanding of the intricate relationship between SLP and employee resilience but also expands our knowledge of the boundary conditions affecting employee resilience. It suggests practical measures to mitigate possible negative consequences of SLP and to foster employee resilience. For instance, increasing organizational goal clarity in the context of SLP can not only extend its positive impact on employee resilience through improved FWA but can also lessen the adverse effects on employee role overload, thereby promoting employees resilience. Future research could further investigate the cross-cultural validity of the relationship between SLP and employee resilience and explore additional boundary conditions that may enhance employee resilience.

## Data Availability

The raw data supporting the conclusions of this article will be made available by the authors, without undue reservation.
